# Thresholds for the Oxford Hip Score after total hip replacement surgery: a novel approach to postoperative evaluation

**DOI:** 10.1007/s10195-017-0465-8

**Published:** 2017-07-06

**Authors:** Nicolai Kjærgaard, Jonas B. Kjærsgaard, Christian L. Petersen, Michael U. Jensen, Mogens B. Laursen

**Affiliations:** 1 Orthopaedic Surgery Research Unit, Department of Clinical Medicine, Aalborg University, Aalborg, Denmark; 2 Department of Orthopaedic Surgery, Aalborg University Hospital, Hobrovej 18-22, 9000 Aalborg, Denmark; 3 School of Medicine and Health, Aalborg University, Aalborg, Denmark

**Keywords:** Arthroplasty, replacement, hip, Osteoarthritis, hip, Patient-reported outcome measure, Patient satisfaction

## Abstract

**Background:**

This is a prospective cohort study to define the thresholds to distinguish patients with a satisfactory or unsatisfactory outcome after total hip replacement (THR) based on patient-reported outcome measures (PROMs) including the Oxford Hip Score (OHS), and using patient satisfaction and patient-perceived function as global transition items. The thresholds are intended to be used as a tool in the process of determining which patients are in need of postoperative outpatient evaluation.

**Methods:**

One hundred and three THR patients who had completed a preoperative questionnaire containing the OHS questionnaire were invited to complete the same questionnaire and supplementary questions at a mean of 6 (4–9) months after surgery. Correlations between outcome measures and anchors were calculated using Pearson’s correlation coefficient. Thresholds were established by receiver operating characteristic (ROC) analysis, using multiple anchors.

**Results:**

Significant correlations were found between outcome measures and anchors. Thresholds were determined for outcome measures coupled with satisfaction, patient-perceived function and a combination thereof using a cut-off of 50 and 70.

**Conclusions:**

We have established a set of thresholds for Oxford scores that may help determine which THR patients are in need of postoperative evaluation. These thresholds can be implemented in clinical practice.

**Level of evidence:**

Level 3.

## Introduction

Traditional quality evaluation of total hip replacements (THRs) includes survival of the prosthesis and revision rates. However, patient-reported outcome measures (PROMs) have recently gained increased attention [1–3]. Joint-specific PROMs allow the assessment of the outcome from the perspective of the patient, including the level of pain and function of the specific joint.

The Oxford Hip Score (OHS) was introduced in 1996 as a measure of postoperative outcome for THR [4]. Used in cohort studies and collected in the national registries [5, 6], it has since been coupled to other patient-reported measures allowing a more comprehensive outcome assessment [1, 2, 6]. This simplifies the interpretation of the quantitative scores into qualitatively meaningful information [7].

Thresholds can be established for OHS values above which patients are satisfied with surgery or have experienced improvement of function after surgery. Defining thresholds for the change value are referred to as the minimal clinically important difference (MCID). Calculating thresholds for the absolute postoperative OHS values, referred to as the patient acceptable symptom state, provides another perspective of patient-perceived outcome [6]. These approaches require the use of global transition items as anchors. Previous studies have used patient satisfaction with surgery and patient-perceived change in function of the specific joint as anchors [1, 2, 6–9].

Recent studies have identified OHS thresholds to aid the clinician in presenting the expected outcome of surgery in a meaningful way to the patient [6]. However, the thresholds may have other possible applications. As the use of the OHS provides a means of comparing preoperative and postoperative health status they may be used as a tool in the process of determining which patients are in need of further postoperative treatment.

In Danish hospitals, there is no standardized method for selecting THR patients in need of postoperative treatment. Current methods range from yearly outpatient visits to nurse-performed telephone interviews using the modified Harris Hip Score [10, 11]. This is very time consuming and the proportion of patients in need of re-evaluation is relatively small and hence does not fully satisfy the time and resources spent. A novel screening system using the OHS as part of a web-based questionnaire was designed as a tool to select patients for outpatient evaluation in the North Denmark Region. Thus, this is a pilot study intended to create an initial algorithm to choose patients in need of outpatient evaluation at 1 year after surgery.

## Materials and methods

Data were obtained from a clinical quality database (“Jointbase”) at the Department of Orthopaedic Surgery, Aalborg University Hospital. The purpose of this database is to monitor the effectiveness of treatment in hip and knee conditions using PROMs. This is assessed through a questionnaire battery using condition-specific instruments (OHS), a generic instrument (EQ-5D-3L) and pain measurements. All patients who completed the questionnaire prior to their surgery and subsequently underwent THR (*n* = 103) in the period between 1 May 2014 and 31 October 2014 were included in the study. Patients were contacted by phone or mail and seen for follow-up in February and March 2015 where the questionnaire was repeated. Additionally, patients completed a postoperative form, which included two global transition items.

As outcome measures, joint-specific PROMs were collected using a Danish translation of OHS for THR patients [12]. As global transition items present overall satisfaction with the outcome of surgery, they were evaluated using a bipolar VAS from 0 (very unsatisfied) to 100 (very satisfied). The present patient-perceived function in the hip compared with before surgery was assessed by a bipolar VAS from 0 (much worse) to 100 (much better).

### Statistical analysis

Descriptive statistics were performed for attenders and non-attenders. The attenders were compared to non-attenders by chi-squared tests for categorical variables and two-sample *t*-tests for continuous variables. To support conclusions of the two-sample *t*-tests, permutation tests were conducted.

Correlations between satisfaction with surgery on the one hand and postoperative OHS or change in OHS on the other hand were calculated using Pearson’s correlation coefficient. Correlations with patient-perceived function were calculated in the same manner.

Using a sensitivity- and specificity-based approach [8], thresholds were calculated for change in OHS (ΔOHS) and absolute postoperative OHS using two global transition items for constructing three anchors—patient satisfaction, patient-perceived function and a combination of the former two using the most conservative value, i.e., the lowest value.

Cut-off points of 50 and 70 for patient satisfaction with surgery were chosen, and thus define a binary outcome; patients with satisfaction values below the cut-off should be invited for out-patient evaluation, and patients with values above the cut-off should not. Likewise, cut-off points of 50 and 70 for patient-perceived function in the hip in question were used. Finally, a set of thresholds was calculated by defining the cut-off as 50 or 70 for the combined anchor. In other words, patients who scored below the cut-off in either one of the two global transition items were identified as patients who should be invited for outpatient evaluation in order to identify and correct reasons for the suboptimal outcome.

Coupling the anchors to the outcome measures (ΔOHS and absolute OHS), sensitivity and specificity for different threshold values were assessed by receiver operating characteristic (ROC) curves plotting sensitivity against specificity. Furthermore, the area under the curve (AUC) was calculated. An AUC between 0.7 and 0.8 is considered acceptable, and an AUC between 0.8 and 0.9 is considered excellent [8]. Thresholds were established for each outcome measure by identifying the point on the relevant ROC curve closest to the upper left corner, as reported by Beard et al. [13].

Calculation of sample size was based on the principles established by Hanley et al. [13], with the α-level (significance) being 0.05, and the β-level (1-power) being 0.20. We wanted to show that an AUC of 0.75 was significantly different to our null-hypothesis (AUC = 0.5), with an expected negative (not called)/positive (called) ratio of 3. This was calculated using MedCalc for Windows, version 17.5.3 (MedCalc Software, Ostend, Belgium).

Statistical analysis was performed using R version 3.1.3 [14].

## Results

One hundred and three patients who underwent primary THR due to primary osteoarthritis in the hip were included in the study. Of these patients, 89 (86.4%) attended the postoperative follow-up at an average of 6.68 (SD 1.7) months after surgery.

No significant differences were found between attenders and non-attenders with regard to gender, age, body mass index (BMI) and preoperative OHS (Table 1, *p* values between 0.12 and 0.22).

**Table 1:** Comparison of preoperative OHS, age, BMI and gender of attenders and non-attenders

	THR patients		
	Attenders (*n* = 89, 86.4%)	Non-attenders (*n* = 14, 13.6%)	*p* value
Preoperative OHS, mean (SD)	20.7 (7.3)	18.5 (6.0)	0.22
Age (years), mean (SD)	67.0 (10.0)	65.7 (15.8)	0.77
BMI, preoperative, mean (SD)	27.8 (6.4)	30.2 (4.0)	0.12
Gender (*n*, %)			1^‡^
Male	41 (46.1)	7 (50.0)	
Female	48 (53.9)	7 (50.0)	

*p* values from two-sample *t* test unless otherwise stated

*SD* standard deviation

^‡^ chi-squared test

OHS increased (mean) by 18.3 (SD 10.4), from 20.7 (SD 7.3) preoperatively to 39 (SD 8.8) after surgery (*p* < 0.01).

We found significant correlations between OHS and patient satisfaction and patient-perceived function, as assessed by simple linear regression and derived Pearson’s coefficient.

Positive correlations were found between satisfaction and postoperative OHS (*r* = 0.73; CI 0.61, 0.81) and between satisfaction and change in OHS (*r* = 0.68; CI 0.55, 0.78).

The same applies to correlations between perceived function and outcome measures. Postoperative OHS (*r* = 0.75; CI 0.64, 0.83) and change in OHS (*r* = 0.61; CI 0.46, 0.73) both show statistically significant positive correlations with perceived function.

To show a significant difference for an AUC of 0.75, thus rejecting the null-hypothesis, the amount of positive cases required was 14, and the amount of negative cases required was 42. This equals a total sample size of 56 cases.

Using a cut-off of 50 for satisfaction, we identified 84.3% (75/89) of THR patients as satisfied. Using a cut-off of 70, we identified 74.2% (66/89) as satisfied.

Patient-perceived function cut-offs of 50 and 70 revealed function gain in 89.9% (80/89) and 79.8% (71/89) of patients, respectively.

The combined anchor identified 82.0% (73/89) of patients as >50 and 71.9% (64/89) of patients as >70.

Thresholds for various outcome measures identified by ROC curves at cut-off values of 50 and 70 for satisfaction and patient-perceived function are presented in Tables 2 and 3.

**Table 2:** Thresholds, percentage of patients who will be called with the given threshold, specificity, sensitivity and area under curve (AUC) for OHS and ΔOHS anchored to patient-perceived satisfaction, function and either satisfaction or function with a cut-off value of 50

Anchor	THR: cut-off value 50				
	Threshold	Called (%)	Specificity	Sensitivity	AUC
Satisfaction (*n* = 14)					
Postoperative OHS	35.5	25.8	0.85	0.86	0.91
ΔOHS	11.5	29.2	0.91	0.71	0.88
Function (*n* = 9)					
Postoperative OHS	30.5	18.0	0.90	0.89	0.83
ΔOHS	15.5	38.2	0.75	0.78	0.87
Satisfaction or function (*n* = 16)					
Postoperative OHS	35.5	25.8	0.88	0.88	0.93
ΔOHS	11.5	29.2	0.92	0.69	0.89

True positives is the amount of patients who should be called according to the cut-off value

*n* number of patients with anchor values below the cut-off value

**Table 3:** Thresholds, percentage of patients who will be called with the given threshold, specificity, sensitivity and area under curve (AUC) for OHS and ΔOHS anchored to patient-perceived satisfaction, function and either satisfaction or function with a cut-off value of 70

Anchor	THR: cut-off value 70				
	Threshold	Called (%)	Specificity	Sensitivity	AUC
Satisfaction (*n* = 23)					
Postoperative OHS	38.5	31.5	0.86	0.83	0.94
ΔOHS	15.5	38.2	0.85	0.74	0.88
Function (*n* = 18)					
Postoperative OHS	38.5	31.5	0.83	0.89	0.92
ΔOHS	17.5	43.8	0.69	0.78	0.83
Satisfaction or function (*n* = 25)					
Postoperative OHS	38.5	31.5	0.88	0.80	0.93
ΔOHS	18.5	44.9	0.69	0.88	0.87

True positives is the amount of patients who should be called according to the cut-off value

*n* number of patients with anchor values below the cut-off value

## Discussion

In summary, we have

- found no difference between patients who attended the postoperative questionnaire and those who did not (*p* values between 0.12 and 0.22),
- found a mean increase in OHS of 18.3 (SD 10.4),
- found significant correlations between OHS and patient satisfaction and patient-perceived function (*r* values from 0.61−0.75),
- established a set of thresholds for absolute postoperative OHS, considering postoperative patient satisfaction and function,
- established a set of thresholds for change in OHS, considering postoperative patient satisfaction and function.

Previous studies have reported mean changes in OHS at 6 months after surgery which is very similar to the findings in this study [6, 7].

Judge et al. found 70.4% of patients reached a satisfactory symptom state based on thresholds for absolute change using a cut-off of 50, and thus 29.6% were grouped as not satisfied. Based on our corresponding threshold, we found the same group to be 29.4%. For the absolute OHS at follow-up, Judge et al. found 26.3% of patients who scored below the threshold. Comparably, we identified 25.8% of patients who scored below the threshold.

We found thresholds for two different outcome measures (absolute OHS and OHS change) using three different anchors and two different cut-offs (50 and 70). This provides additional perspectives and a better foundation for evaluating the different strengths and limitations of each threshold before the actual use as thresholds for contacting patients. In line with previous studies [2, 6, 7], we were able to document significant correlations between the global transition items (satisfaction and patient-perceived function) and both outcome measures (*r* values from 0.61−0.75), justifying the use of these as anchors when establishing thresholds for the outcome measures.

Using a cut-off of 50 for each anchor, we established thresholds for change in OHS and postoperative OHS. The thresholds found in this manner were shown to have reasonable levels of sensitivity and specificity and to be consistent with results presented by Judge et al., thus supporting these findings.

It may be questioned whether a cut-off of 50 is appropriate in this setting. Given the phrasing of the question, a VAS score of 50 indicates indifference concerning satisfaction and lack of change in function. Thus, choosing a cut-off of 50 to discriminate between patients satisfied and not satisfied implies the assumption that all patients who are more than indifferent, are indeed satisfied. We aim for patients to be more than just above “indifferent” after having surgery. Similarly, patients with a function perception of 50 are not experiencing a change in function. With that in mind, we added a higher cut-off (70) to our analysis in order to detect patients who might have a suboptimal surgery outcome. By introducing a cut-off of 70, another set of thresholds was calculated detecting a larger proportion of patients for out-patient evaluation.

Previous studies have focused on one global transition item and OHS, thus using a simpler approach to define thresholds for satisfactory surgery outcomes. This may leave out potentially important perspectives which this study aims to accommodate by including two different global transition items. The purpose of previous studies has been to provide clinicians with simple and meaningful information regarding outcome after surgery and at the same time allowing a more comprehensive interpretation of the OHS. Our results may be used in the same fashion, although this has not been the main aim of our study. We have provided a large body of limits potentially useful in the clinical process of choosing patients for postoperative evaluation.

The follow-up time is an average of 6.68 months after surgery. This may raise the question of whether the patients have reached their steady state of improvement on the OHS or not. A systematic review on the matter found some evidence that patients may not yet be in a steady state after six months; however, the study did not establish a definitive conclusion [15]. We acknowledge it would have been preferable to base the study on patients at 12 months after surgery. However, as this project is intended to create an initial algorithm for a novel system, and there were no patients in the database who had yet reached the date for a 1-year follow-up, we believe the theoretical improvement in OHS does not change the value of the results.

A concern regarding implementation of our thresholds as stand-alone criterions for postoperative evaluation is the considerable number of patients not in need of postoperative evaluation who are identified by the established thresholds because of specificity values <1. This could be accommodated by an additional filter, e.g., interviewing the identified patients by phone beforehand to minimize the number of unnecessary consultations.

The sample size of 89 THR patients is relatively small compared to other studies including hundreds or thousands of patients [1, 6, 7]. As addressed previously, there is consistency between our results and those of previous studies. This supports the assumption that our results are representative of the population.

However, as a consequence of the relatively small cohort, adjusting for confounding factors between attenders and non-attenders was not found relevant. Furthermore, the absolute number of patients classified as eligible for evaluation is relatively low (9–25 patients). Thus, small differences in outcome measures for these patients would have a large impact on the established thresholds. This made it impossible to yield meaningful results if patients were stratified according to age, preoperative scores, etc. This approach would be preferable as it would have been possible to define differentiated thresholds based on the preoperative OHS. An alternative to stratification of patients according to preoperative scores is calculating thresholds for the percentage of potential change [3]. This takes into account the maximum increase possible for each patient. As the scores range from 0−48, the OHS includes a ceiling effect, meaning that patients with a higher preoperative score have a lower potential of change than patients with a low preoperative score. As an example, if a threshold for change in the OHS of 15.5 points is used as the only limit, patients with a preoperative score of 10 will not be called if their postoperative score is >25 points. However, patients with a preoperative score of 30 points will be called despite scoring 45 points, which is close to the maximum score of 48 points. Furthermore, patients with a preoperative score of ≥34 will inevitably be called for evaluation, because their maximum possible improvement is 14 points. Thresholds for absolute OHS involve a comparable problem as patients with a relatively low preoperative OHS may have a large and satisfactory improvement but still not reach the threshold.

Considering the global transition item regarding change in function, one may argue that recall bias could be a problem. However, our main interest is the patient’s own experience of function change at the present time. Thus, we recognize the possibility that patients are not fully capable of remembering the exact state of function before surgery, but since this is not our main concern we believe that this does not constitute a problem.

Judge et al. [6] have shown a variance in thresholds for postoperative scores and change of OHS anchored to satisfaction when stratifying patients according to preoperative scores. Further research on larger sample sizes may establish an array of thresholds based on patient groups stratified by preoperative OHS and other variables. This may allow the use of these thresholds as decisive for calling patients for evaluation, thus eliminating the need for the additional filter proposed previously.

In summary, we have established a set of thresholds for the OHS that discriminates between patients who are satisfied with THR surgery at 6 months postoperatively and patients who are not. A similar set of thresholds has been established to differentiate between patients who have or have not experienced a gain in function after surgery.

The set of thresholds presented in this study may be used when choosing limits in a system, which determines whether or not to call patients for postoperative evaluation, based on at-home web-based questionnaires including OHS. These thresholds may require the use of an additional filter to detect patients not in need of evaluation.

To establish thresholds applicable as sole determinants of which patients should be offered postoperative evaluation, we advise further research on larger sample sizes, allowing stratification of patients.
